# Acute Toxicity of Aqueous Extract from *Bredemeyera floribunda* Root Bark in an Animal Model

**DOI:** 10.1155/2024/8991384

**Published:** 2024-06-24

**Authors:** Cláudia Stela Medeiros, Beatriz Medeiros, Maria Lígia Macedo, Rita Guimarães, Karine Freitas, Danielle Bogo, Priscila Hiane, Ricardo Viana, Valter Nascimento

**Affiliations:** ^1^Saúde e Desenvolvimento da Região Centro-Oeste, Universidade Federal de Mato Grosso do Sul, Campo Grande, Brazil; ^2^Faculdade de Medicina, Universidade Federal de Mato Grosso do Sul, Campo Grande, Brazil; ^3^Laboratório de Anatomia, Instituto de Educação Física e Esportes, Universidade Federal do Ceará, Fortaleza, Brazil

## Abstract

The medicinal plant *Bredemeyera floribunda Willd*. is used to treat cardiovascular disease, chronic fatigue, low libido, as well as increased diuresis. However, studies considering the toxicity of this plant are scarce. Develop an aqueous extract of *B. floribunda* considering traditional use and determine the average lethality (LD_50_), signs, and symptoms of toxicity. The *B. floribunda* extract was obtained by immersing the root bark in ultrapure water for 18 hours at 4°C, under constant stirring. The test extract was administered in a single dose of 2.000 mg/kg by gavage to rats. Signs and symptoms of toxicity were determined according to the Hippocratic screening test and compared with the control group. In addition, a necropsy was performed for macroscopic evaluation of the organs in the abdominal cavity. A powder was obtained from aqueous extracts that showed the same organoleptic characteristics and emulsification capacity as those presented by the fresh root when prepared according to popular tradition. The LD_50_ was greater than the test dose with three animals surviving. On the other hand, necropsy of dead rats showed necrosis and reduction in lung mass, in addition to the presence of foam and excessive distension of the stomach and intestines. The main symptoms of toxicity were anesthesia, ataxia, sedation, loss of muscle strength, and excessive drowsiness in the first 24 hours. There was no difference between the control and extract groups with regard to body mass, food, and water intake, as well as in macroscopy of the heart, liver, lungs, intestines, spleen, pancreas, and kidneys. The aqueous extract of the *B. floribunda* was considered nontoxic or of very low toxicity. However, it is capable of altering the activity of the central nervous system and causing disorders in the respiratory and digestive systems.

## 1. Introduction

According to WHO, there has been an increase in the consumption of medicinal plants in several countries [1, 2]. This increase in recent years is attributed to the low cost of medicinal plants, accessibility, and the belief that medicinal plants do not cause harm to health [3, 4]. In an effort to ensure human health, some countries have developed guides, laws, and regulations containing the botanical identification and commercialization of phytomedicines [1, 5], in addition to encouraging research on the identification of the physical compounds of medicinal plants [6, 7].

The *Bredemeyera floribunda* Willd. (Polygalaceae) is a medicinal plant native to the tropical Americas and Australia [8]. In Brazil, it is popularly known as *botica inteira*, *cipó-gemada*, *laça-vaqueiro*, *pau caixão*, *João da Costa*, raiz de *cobra*, *pacari*, and *pau de ovo* [9–11]. Parts of this plant can be prepared in the form of decoction, infusion, maceration, and as juice, having applications in the treatment of diseases such as high blood pressure, liver, lung, and cardiovascular diseases [12–14], insect bites, snakes, and gynecological diseases [15, 16]. In addition, in popular medicine, the bark of the root of *B. floribunda* is used against fatigue and exhaustion, increasing work activity and libido [17]. The root bark, when scraped and mixed with water, produces an aerated cream with a firm texture, which is ingested daily in the first meal of the day in rural areas [17, 18].

The popular use of *B. floribunda* has been proven through experiments using the ethanol extract of the root, in which it has been shown to be effective in protecting the gastric mucosa in rats [10]. In addition, mice studies demonstrated antiophidic activity against the pit viper venom (*Bothrops jararaca*) [15, 19]. However, the popular use of this plant as a hypotensive has not been confirmed [13].

The chemical extraction methods for obtaining medicinal plant extracts and the type of organic solvents used (ethanol, methanol, and acetone, among others) directly influence the concentration of secondary metabolites and other bioactive compounds present in the final product [20–22]. However, such methods do not reproduce the prevalent popular use, which in turn uses plants in the form of teas [17], juices and macerates; or immersion of dried or fresh plant parts in water [23, 24]. In this sense, components such as saponins, xanthones, rutin, cinnamic acid, and flavonoids [19, 25, 26], as well as the already elucidated effects and actions of the root of *B. floribunda*, refer to the ethanolic extract [10, 11, 14, 15]. However, the effectiveness and adverse effects of the root bark obtained according to popular water management methods are still scarce in the literature.

Since there is a scarcity of information on the popular use of the aqueous extract of the bark of *B. floribunda,* experimental models are important to stipulate the limit dose, as well as the adverse effects or harmlessness for oral use of the popular tradition, which is the baseline reference for conducting clinical trials [2, 5, 7]. Therefore, this study aims to develop a method of aqueous extraction, mimicking the form of popular consumption, as well as to carry out a toxicity test using an animal model.

## 2. Methods

### 2.1. *Bredemeyera floribunda* Willd. Plant Collect

The roots of *B. floribunda* were collected from “*Assentamento Bebedouro*,” in *Nova Alvorada do Sul* city, *Mato Grosso do Sul* state, Brazil (21 38′80.67″S–54 43′63.93″W) in November 2020. The removal of roots was in the afternoon, the relative humidity was at 56%, and the temperature was 34.3°C [27]. To preserve the integrity of the roots, a circular area of approximately 25 cm was delimited around from the stem. After careful excavation, the roots were removed from the ground and sectioned from the stem, kept wrapped with soil as a means of protection against the incidence of the sun's rays, and packed in plastic packaging, followed by packaging in a thermal box (0°C–5°C) for transport to the laboratory. The plant was deposited in the Biology Herbarium/Federal University of Mato Grosso do Sul under the number 54366 CGMS and was registered in the National System for Management of Genetic Resources and Associated Traditional Knowledge (SisGen, A7716EC).

### 2.2. Extraction Extract of the Root Bark

In this study, an aqueous extract was obtained considering traditional popular knowledge, with the purpose of preserving the characteristics of the fresh plant, minimizing oxidation, loss of nutrients, and secondary metabolites. All experiments were carried out in the laboratory at a temperature of 18°C to 20°C and without artificial lighting [28–30]. We initially washed the roots of the *B. floribunda* with abundant ultrapure water (18.2 MΩ, Purelab® Option Q15—Elga; Brazil) and then placed them in a white polyethylene basin. In sequence, a soft bristle brush and ultrapure water were used to gently brush the surface and remove the dirt that was adhered to the *B. floribunda* roots [31]. Then, the root bark was removed from the wood using a scalpel. Root samples were packaged in-film bags, then wrapped with aluminum foil, and stored at −80°C. The starts of the extraction process were carried out in the following sequence:

1. Protect against the light, the sample was thawed at a temperature of 20°C for 1 hour.
2. 52 grams of bark roots were weighed and then macerated in liquid nitrogen to disrupt the plant tissues and preserve the heat-volatile analytes [32].
3. The root bark macerated was transferred to a beaker and was added to 468 mL of the ultrapure water, obtained an aqueous solution in a ratio of 1 to 9 (1 : 9).
4. The bark-root extract aqueous solution remained on a magnetic stirrer at speed 4 (Fisatom® 751, Brazil) for 18 hours and inside a refrigerator (4°C; Springer®, Brazil).
5. The bark-root extract aqueous solution was centrifuged at 1500 rpm for 30 minutes at 4°C (Hitachi® CR 22 GIII, Japan).
6. The supernatants (bark-root extract aqueous solution) were immediately frozen (solução etilenoglicol; Christ CB 18–40) starting the freeze-drying process until a powdered aqueous extract was obtained (Freeze dryer, Jotur® L101. Brazil).
7. The powdered aqueous extract is packaged against light and stored at −80°C until the acute oral test is carried out.

All experiments described above were developed and submitted to analysis of patent in Brazil in with registration number as BR 1020210170735 [33].

### 2.3. Animal and Experimental Design

All experiments were only performed after the approval by the Animal Research Ethics Committee of the Federal University of Mato Grosso do Sul (CEUA/UFMS no. 1147/2020), according to the Animal Research: Reporting of In Vivo Experiments (ARRIVE 2.0) [34], and the National Animal Experiment Control Council [35]. Wistar (*Rattus norvegicus*) female adult rats (*n* = 10, 56 days of age and 170.31 ± 12.23 g) were allocated in two groups (control or experimental) with five animals each [36, 37]. The selection criteria for the animal model were used for choosing the animal model according to the 425 OECD protocol [38] and Manole and Robichaud [36].

To realize the acute toxicity testing, the female rats nulliparous and nonpregnant were housed by the SNOSE (sequentially numbered opaque sealed envelopes) method [39, 40] in collective cages at 21°C ± 2°C on a 12 : 12 light-dark cycle and received food (Nuvilab®) and water *ad libitum.* Initially, there was familiarization of all the female rats with the management for determination of body mass and specific needle gavage procedure for rats. After eight days, the animals were submitted to overnight fasting to perform the oral acute toxicity test, and the *Hippocratic screening test* was applied to monitor symptoms for the next 14 days [36]. After 14 days, the animals were anesthetized with xylazin and ketamin (intraperitoneally, 0.06 mL/100 g/BM and 0.03 mL/100 g/BM, respectively) and submitted to euthanasia by exsanguination. The heart, lung, liver, spleen, pancreas, kidneys, and intestines were removed, weighed, and analyzed macroscopically to verify the presence of possible alteration [41].

### 2.4. Toxicity (LD_50_) and Hippocratic Screening Test

The AEBF acute oral toxicity test was performed according to the OECD-425 guideline, and the toxicological effect was evaluated based on mortality and expressed as LD_50_. A test dose value of 2.000 mg/kg was considered in this study in accordance with the OECD 425 protocol. Thus, the test dose of 2000 mg/kg was diluted in 1.0 mL·kg and administered with an intragastric gavage needle to rats, respecting an interval of approximately 20 minutes between the animals in the EG. Concomitantly, the CG animals received 1.0 mL·kg of ultrapure water [36–38].

To carry out the toxicity test (DL_50_), the AEBF was removed from the ultrafreezer and exposed to a temperature of 21°C ± 3°C for two hours. Then, the opaque packaging was removed, the extract was diluted in ultrapure water, and the specific dose was infused into each animal.

The observational analyses of the clinical symptoms and the organs were performed according to the adapted *Hippocratic screening test* (Malone and Robichaud [36]). The behavioral changes of the animals were recorded according to six axes as follows: (i) assessment of the conscious state, disposition, and general conditions; (ii) assessment of the activity and lack of coordination of the motor system; (iii) assessment of skeletal muscle activity; (iv) assessment of auricular and corneal reflexes; (v) assessment of the central nervous system (CNS) activities; and (vi) assessment of autonomous activity and the clinical symptoms' evolution were recorded on a scale from zero to four according to the absence or intensity of behavioral symptoms.

Tests to analyze toxicity symptoms were performed before and after gavage with the EABF. Animal evaluations after inoculation of the thess doses were recorded after 30 minutes (1^st^ evaluation), 1 hour (2^nd^ evaluation), 2 hours (3^rd^ evaluation), 3 hours (4^th^ evaluation), 4 hours (5^th^ evaluation), 5 hours (6^th^ evaluation), 6 hours (7^th^ evaluation), 12 hours (8^th^ rating), and 24 hours (9^th^ rating). From the 8^th^ assessment onwards, analyses occurred once a day and at 8 a.m. for two weeks.

### 2.5. Statistical Analysis

Data normality was tested using the Shapiro–Wilk test. As data did not present a normal distribution, Friedman's test was used to compare animals' body mass measured at six different time points: before familiarization, before fasting, immediately postfasting, one day after infusion, seven days after infusion, and 14 days after infusion. When necessary, Conover's' post-hoc test adjusted by the Bonferroni post-hoc test was used to identify paired differences. Kendall's W was used as the effect size for the Friedman test. Kendall's W concordance degree was classified as “no agreement” (*W* < 0.10), “weak agreement” (0.10 ≥ *W* < 0.30), “moderate agreement” (0.30 ≥ *W* < 0.60), “strong agreement” (0.60 ≥ *W* < 1.0), and “perfect agreement” (*W* = 1) [42]. The Mann–Whitney *U* test was used to compare differences in body mass between the experimental and control groups at each time point. Rank-biserial correlation (rB) was adopted as an effect size measure for the Mann–Whitney test. The rB values were classified according to the Pearson coefficient of correlation as “trivial” (rB < 0.10), “small” (0.10 ≤ rB < 0.30), “medium” (0.30 ≤ rB < 0.50), and “large” (rB ≥ 0.5) [43, 44]. Parametric tests were not performed as the available data did not meet the requirements (e.g., homogeneity of variance), even though after data transformation to logarithm. Data are presented as the median and interquartile range (IQR) and median difference and 95% confidence interval (CI). All data were analyzed using Jeffreys's Amazing Statistics Program (JASP, version 0.17.3.0, Netherlands), and a significance level of 0.05 was set for all statistical tests.

## 3. Results

### 3.1. Aqueous Extracts of Plants

**Figure 1:**
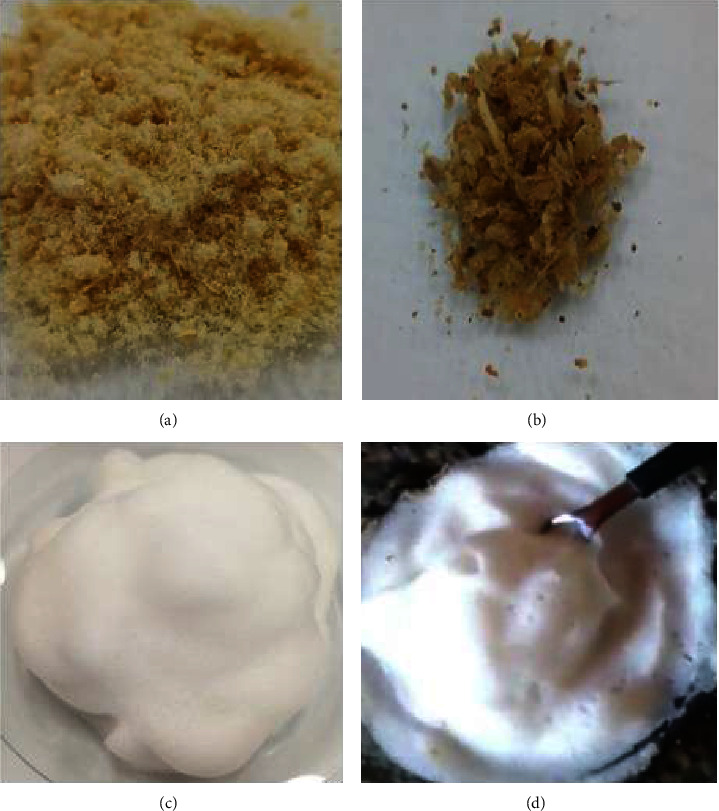
(a) Aqueous extract obtained from the fresh bark of *Bredemeyera floribunda Willd* (EABF) according to popular knowledge, (b) specimen of fresh shaved root bark, (c) cream obtained after vigorously mixing the EABF obtained with water, and (d) aerated cream manipulated according to popular tradition.

AEBF was solid in nature (powder) and had organoleptic characteristics similar to those of the medicinal plant of traditional popular use (Figure 1).

### 3.2. Determination LD_50_ and Necropsy

**Figure 2:**
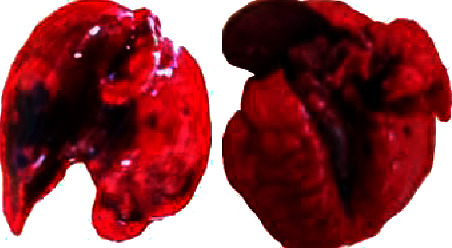
Lungs of rats killed 24 hours after intragastric gavage with an aqueous extract of *B. floribunda*.

**Figure 3:**
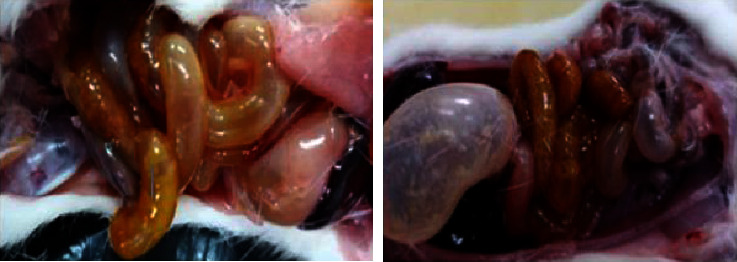
Specimen from the abdominal cavity of rats killed in the first 24 hours of the EABF acute oral test.

The LD_50_ from EABF was greater than 2.000 mg/kg, resulting in the death of two rats. The necropsy performed allowed the identification of anatomical changes in the lungs (Figure 2), stomach, and intestines (Figure 3). According to the necropsy of the surviving animals carried out at the end of the toxicity test, there were no macroscopic differences in the organs of the surviving animals in relation to the control group.

### 3.3. Signs and Symptoms of Toxicity from the Hippocratic Screen

#### 3.3.1. Assessment of Conscious State, Disposition, and General Conditions

From the comparison of the results of the control groups with the extract group, changes were found in the conscious state and disposition in the GE in the first 60 minutes after the EABF gavage, as well as respiratory changes (tachypnea), hypnosis, sedation, deep sleep, numbness (anesthesia), exacerbated drowsiness, cyanosis of the extremities, and absence of sound noises. However, these symptoms gradually and progressively reduced after 12 hours of inoculation with the EABF aqueous solution. Behaviors between the control and experimental groups became similar from the first 24-hour assessment.

There were no differences between the control and experimental groups in relation to general conditions such as fur, paw skin, tail and inner part of the ears, mouth, and teeth; however, there were nosebleeds presented by two rats in the experimental group in the first 30 minutes.

#### 3.3.2. Assessment of Activity and Incoordination of the Motor System

Ataxia was identified only in the experimental group, as well as the absence of spontaneous movements and responses to stimuli inducing reflex reactions. After five hours, there was no difference between signs and symptoms of intoxication in the control and experimental groups.

#### 3.3.3. Assessment of Skeletal Muscle Activity

According to the assessment of skeletal muscle activity, during the first four hours after gavage, the animals of the experimental group were unable to support their body mass on the test grid, regardless of the angle at which the grid was arranged or the intensity of the shaking that was performed. On the other hand, there was a return of moving and muscle strength gradually over 12 hours, becoming similar to the control group after this time.

#### 3.3.4. Assessment of Auricular and Corneal Reflexes

According to the results of the evaluation of auricular and corneal reflexes, the EG group did not respond to the evaluation tests during the first four hours. However, after this time, the experimental and control groups responded equally to the tests applied.

#### 3.3.5. Assessment of Central Nervous System (CNS) Activities

The results of the CNS evaluation tests in the animals showed hypnosis, sedation, and anesthesia, which were gradually reduced during the first 4 hours of analysis. However, the animals in the experimental group remained drowsy than those in the control group for 24 hours. No convulsions, tail erection or the Straub response, or other symptoms indicative of CNS toxicity were observed.

#### 3.3.6. Assessment of Autonomic Activity

**Figure 4:**
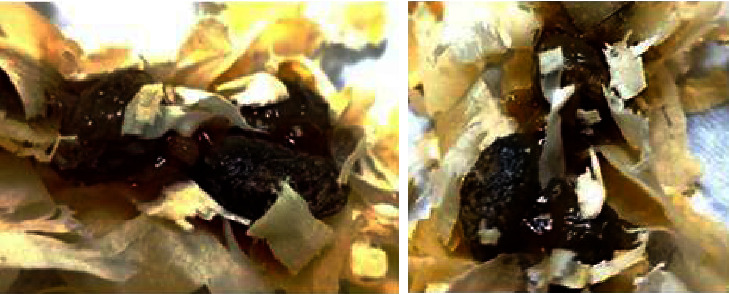
Samples of black stool containing mucus and traces of blood from two rats in the extract group.

No traces of urine were observed during the first three hours, and evacuation was for four hours. However, after five hours, the EG group's stools were found to be blackish, a color that remained until the second day of the evaluation tests. Furthermore, there were blood and mucus covering the fecal samples from two rats (Figure 4), which died before the first 24 experimental hours. There was no difference between the control and experimental groups regarding symptoms such as tearing, salivation, ptosis, and piloerection.

### 3.4. Body Mass

**Figure 5:**
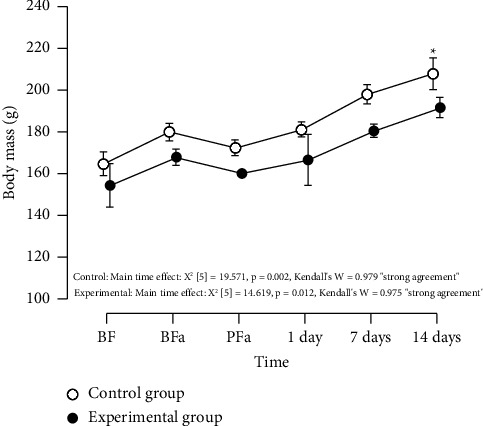
Descriptive plot presenting experimental and control group rats' body mass over the intervention. BF: before familiarization. BFa: immediately before fasting. PFa: immediately postfasting. 1 day: 1 day after extract infusion. 7 days: 7 days after extract infusion. 14 days: 14 days after extract infusion. g: grams. ^*∗*^*p*=0.048 (BF vs. 14 days). There were no significant differences (*p* > 0.05) in the animals' body mass between the experimental and control groups for all time points. Note: data are presented as the mean and 95% confidence interval.

**Figure 6:**
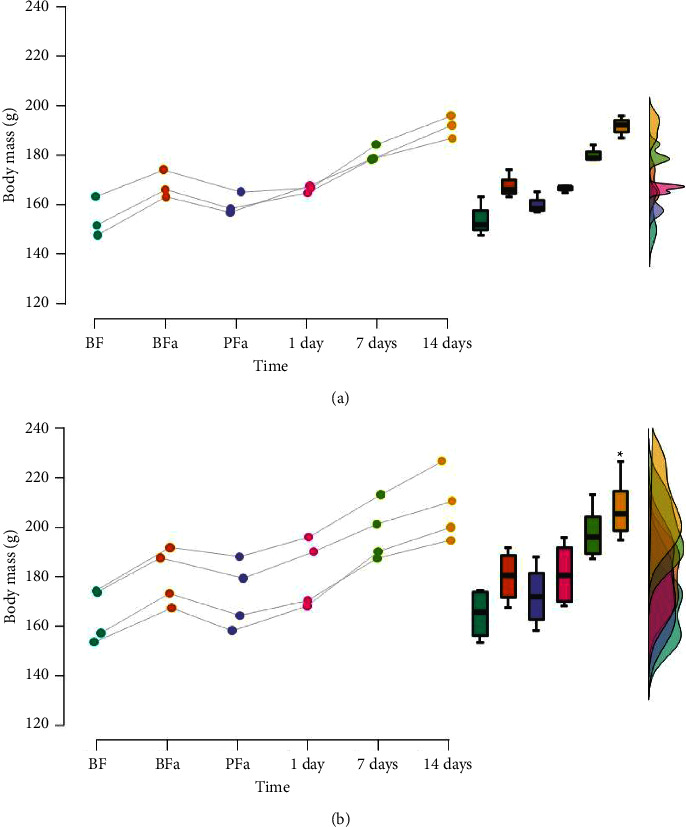
Raincloud plots presenting (a) experimental and (b) control group rats' body mass over the intervention. BF: before familiarization. BFa: immediately before fasting. PFa: immediately postfasting. 1 day: 1 day after extract infusion. 7 days: 7 days after extract infusion. 14 days: 14 days after extract infusion. g: grams. ^*∗*^*p*=0.048 (BF vs. 14 days). There were no significant differences (*p* > 0.05) in the animals' body mass between the experimental and control groups for all time points. Note: the dots represent individual data for each time point.

Friedman's test showed a significant time effect on rats' body mass in the experimental group (*χ*^2^ [5] = 14.619, *p*=0.012, Kendall's *W* = 0.975 “strong agreement”); however, pairwise comparisons showed no significant differences in female rats' body mass between any time points (*p* > 0.05, Figures 5 and 6(a)). Friedman's test showed a significant time effect rats' body in the control group mass (*χ*^2^ [5] = 19.571, *p*=0.002, Kendall's *W* = 0.979 “strong agreement”). Pairwise comparisons showed that rats reported a significant increase in body mass only 14 days after extract infusion (*p*=0.048) compared to the before familiarization time point; however, body mass did not differ between the other time points (*p* > 0.05, Figures 5 and 6(b)). The Mann–Whitney test showed no significant differences (*p* > 0.05) in the body mass of female rats between experimental and control groups for all time points (Table 1).

### 3.5. Organ's Weight

Regarding the organ weight, only the pancreas of the female rats allocated to the experimental group (median: 0.33 [IQR: 0.01]) was significantly heavier (median difference: −0.07 [95% CI: −0.09; −0.06], *p*=0.048, “large effect”) than those female rats allocated to the control group (median: 0.26 [IQR: 0.01]).  Table 2 presents additional information about the organ weight of the animals from experimental and control groups.

## 4. Discussion

The aqueous extract obtained from the bark of the *B. Floribunda* root (AEBF) presented a solid nature (powder) (Fig. A), with aroma and emulsion potential equal to those consumed by popular tradition (Fig. C and D); however, the acrid flavor was more intense, masking the initial sweet flavor and promoting immediate salivation. According to Wasicky [11], the intense acrid flavor is a characteristic of the saponins present in the roots of *B. floribunda*.

The AEBF color showed a light-yellow brown color and the presence of visible shiny crystals, while the fresh root peels had a dark, opaque yellowish-brown color (Fig. B). According to Judith [9], the crystals present in these plants are calcium oxaloacetate, which is found in greater quantities in the bark than in the root wood. In this way, our results showed that it was possible to obtain the extract and aerated cream, mimicking the popular tradition used to treat chronic fatigue, excessive tiredness, and low libido, as described by Tschinkel et al. [17].

Although this plant is used by the traditional population, there are few clinical reports on its lethality [15]. Therefore, it was decided to determine the LD_50_ and toxicity symptoms using a single dose of 2000 mg/kg [38], which has been widely used to classify the lethality of medicinal herbs in use by popular tradition [37, 45]. Thus, in the survival of three rates, out of the total of five that received the test dose, the LD_50_ of EABF was classified as up regulation or greater than the test dose [38], therefore, with low toxicity and safe for human ingestion [46, 47].

According to the World Health Organization [2], traditional medicine has become a global phenomenon, and about 80% of the population in developing countries depends almost totally on herbal medicine for their primary health care needs. Thus, it is critical to carry out acute toxicity tests to elucidate the toxicity or otherwise/nontoxicity of plants in use by popular tradition but unknown in terms of health risks. In this sense, Sasso et al., [37], who classified as nontoxic the aqueous extract obtained from the leaves of *Annona muricata* Lin. In other study, the dose of 2.000 mg/kg of the ethanolic extract obtained from the rhizomes of medicinal plants *Kaempferia galanga*, that administered intraperitoneally caused the death of two out of every four rats within 24 hours, in addition to analgesia, decreased motor and respiratory activity such as signs and symptoms of toxicity. However, this same extract when administered orally at a dose of 5.000 mg/kg appeared to be nontoxic. In general, the toxicity of medicinal plants seems to depend on the part of the plant used, as well as the method used to obtain the extract [45].

The results obtained in our LD_50_ study corroborated those presented by Alves et al. [15], who obtained the same LD_50_ for the ethanolic extract of the *B. floribunda* root, however, without animal deaths. The occurrence of two animal deaths in our experiment can be attributed to the fact that saponins are more soluble in water than in alcohol [9, 11]. In addition, previous studies have shown that the effect of saponins is dose-dependent [10, 13, 48].

The necropsy of the two rats showed anatomical changes in the stomach and intestines, which were excessively distended, compressing the other organs of the abdominal and thoracic cavity (Figures 2 and 3). In addition, a reduction in lung mass volume, irregular contours of the parenchyma, and necrosis were observed, which are common changes in pulmonary complications such as atelectasis [49, 50], and shunt pulmonary disease due to occlusion of the superior vena cava (Figure 3) [51], which can lead to death [49, 51, 52]. Organ distension (Figure 3) may be caused by the emulsification of saponins, as the presence of a large amount of “foam” was observed in the gastrointestinal lumen.

According to the results, there are no macroscopic changes in the heart, spleen, and pancreas. Furthermore, the results of the necropsies performed during euthanasia showed that there was no difference between the control and extract groups in relation to the morphology and weight of the organs, heart, lungs, spleen, liver, kidneys, and intestines (Table 1) [36, 38]. However, the mass of the EG pancreas was greater than the mass of the CG. According to Liu et al. [53], saponins in some plant species can activate the pancreatic hypertrophic (PI3K/Akt) pathway, causing significant changes in this organ.

Regarding signs and symptoms of toxicity, behavioral changes began approximately 15 minutes after administration of AEBF (337.8 ± 24.6 g). The animals suddenly and abruptly became agitated, followed by short breathing (tachypnea), glassy gaze, staggering gait, drowsiness, deep sleep, sedation, and anesthesia. In addition to hypnosis, loss of muscle strength, reduced motor activity, anuria, and transient constipation. Furthermore, according to our observation, the breathing of the animals in this group remained short and accelerated for approximately 1 hour, with nasal bleeding being identified in two animals.

Nasal bleeding can be explained because of the greater solubility of saponins in water than in the extractants used to determine the hemolytic activity of *B. floribunda*. Furthermore, the low digestibility, low bioavailability, and high stability of the *B. floribunda* emulsion [54] are capable of extending the activity time of this secondary metabolite and contributing to the several exacerbated effects found [55–57].

In the present study, the signs and symptoms of toxicity, such as symptoms of anesthesia, hypnosis, sedation, and deep sleep, and the reduction in motor activity and muscle strength, are similar to those that attest to the effectiveness of phytomedicines rich in saponins in the treatment of depression, anxiety, and mental disorders, sleep, and neurodegenerative diseases [48, 58, 59]. Furthermore, saponins have been studied for their sedative, anesthetic, and opioid effects, with a confirmed effect on neurotransmitters, cytokines, and hormones related to sleep [60].

Symptoms of gastrointestinal toxicity were not found with the same intensity and duration of time as those presented here (absence of evacuation, gastric distension, mucus, and blood coating the blackened feces. On the other hand, Yao et al. [48] observed a reduction in fecal production and defecation; the time for symptoms to appear was only 60 minutes after administration of saponins from *Polygala tenuifolia*. However, gastric changes are common complaints in studies using phytomedicines obtained from medicinal plants such as Polygalas [59, 61–63].

The clinical observations in our study using an animal model coincide with those described by the population who report that the use of this plant can cause softening in the legs, anguish, heart pounding, diarrhea, or constipation. The AEBF did not change daily feed consumption, as well as water and body mass, which remained in the standard for rodents [64, 65], thus corroborating the results of Arun and Asha [66], who used the same variables to classify the lack of toxicity of the crude aqueous extract obtained from the medicinal plant *Physalis peruviana* Linn., which is rich in saponins.

According to the results obtained in our studies, although AEBF did not present toxicity, the symptoms involving the animals revealed the manifestation of transient toxicity symptoms. Due to the possibility of adverse effects, we strongly recommend further studies to better understand the mechanisms of action of AEBF.

## 5. Conclusion

The aqueous extract obtained from the bark of the *B. Floribunda* root presented a solid nature (powder) and light-yellow brown color, and aroma, as well as the presence of visible shiny crystals, and emulsion potential equal to those consumed by popular tradition.

The LD_50_ of EABF was classified as up regulation, therefore, with low toxicity and safe for human ingestion. The results obtained in our LD_50_ study corroborated those presented by the literature who obtained the same LD_50_ for the ethanolic extract of the *B. floribunda* root. The necropsy of the two rats showed anatomical changes in the lungs, stomach, and intestines, which were excessively distended. There was no difference between the control and extract groups in relation to the morphology and weight of the organs, heart, pancreas, lungs, spleen, liver, kidneys, and intestines. However, the animals showed transient signs and symptoms of toxicity.

The current study presents information on the acute toxicity of the aqueous extract of *Bredemeyera floribunda* roots, serving as an initial evaluation of the extract's safety. For recommendations on the clinically safe dose, a repeated dose toxicity study is imperative. Lastly, it is necessary to carry out new studies to investigate the secondary metabolites and mechanisms of action capable of generating changes identified in the CNS, respiratory, and gastrointestinal.

## Figures and Tables

**Table 1:** Comparison of rats' body mass (g) between the experimental and control groups at each time point of the study period.

Time point (g)	Experimental group (*n* = 3)		Control group (*n* = 4)		Median difference (95% CI)	*p* ^*∗*^	Effect size	
	Mean ± SD	Median [IQR]	Mean ± SD	Median [IQR]			*r*_*B*_ (95% CI)	Classification
BF	154.23 ± 8.10	151.70 [7.80]	164.65 ± 10.85	165.40 [17.50]	9.90 (−9.80; 26.60)	0.229	0.67 (−0.12; 0.94)	Large
BFa	167.82 ± 5.69	166.06 [5.48]	179.98 ± 11.55	180.37 [16.88]	11.67 (−6.75; 28.54)	0.229	0.67 (−0.12; 0.94)	Large
PFa	160.12 ± 4.45	158.35 [4.18]	172.44 ± 13.64	171.78 [18.62]	10.77 (−7.00; 31.20)	0.400	0.50 (−0.36; 0.90)	Large
1 day	166.46 ± 1.51	166.91 [1.46]	181.15 ± 13.92	180.19 [21.70]	13.96 (0.57; 31.19)	0.057	1.00 (1.00; 1.00)	Large
7 days	180.46 ± 3.39	178.63 [3.00]	197.93 ± 11.77	195.65 [14.83]	14.27 (2.94; 34.71)	0.057	1.00 (1.00; 1.00)	Large
14 days	191.66 ± 4.63	192.08 [4.62]	207.90 ± 14.10	205.15 [15.90]	13.70 (−1.40; 39.80)	0.114	0.83 (0.27; 0.97)	Large

IQR: interquartile range. SD: standard deviation. CI: confidence interval. BF: before familiarization. BFa: immediately before fasting. PFa: immediately postfasting. 1 day: 1 day after extract infusion. 7 days: 7 days after extract infusion. 14 days: 14 days after extract infusion. g: grams. ^*∗*^*p* values from the Mann–Whitney *U* test. *r*_*B*_: rank-biserial correlation.

**Table 2:** Comparison between control and experimental rats' organ weight after the study period.

Organs (g)	Experimental group (*n* = 3)		Control group (*n* = 4)		Median difference (95% CI)	*p* ^*∗*^	Effect size	
	Mean ± SD	Median [IQR]	Mean ± SD	Median [IQR]			*r*_*B*_ (95% CI)	Classification
Liver	8.73 ± 1.51	7.91 [1.34]	8.85 ± 1.24	8.40 [1.13]	0.18 (−2.52; 2.84)	0.400	0.50 (−0.36; 0.90)	Large
Pancreas	0.33 ± 0.01	0.33 [0.01]	0.25 ± 0.01	0.26 [0.01]	−0.07 (−0.09; −0.06)	0.048	−1.00 (−1.00; −1.00)	Large
Spleen	0.43 ± 0.03	0.44 [0.03]	0.47 ± 0.03	0.46 [0.02]	0.03 (−0.02; 0.11)	0.226	0.58 (−0.25; 0.92)	Large
Lungs	1.20 ± 0.21	1.17 [0.21]	1.13 ± 0.10	1.15 [0.10]	−0.03 (−0.43; 0.21)	0.857	−0.17 (−0.80; 0.64)	Small
Heart	0.62 ± 0.06	0.59 [0.06]	0.69 ± 0.06	0.69 [0.05]	0.09 (−0.07; 0.19)	0.229	0.67 (−0.12; 0.94)	Large
Right kidney	0.75 ± 0.12	0.71 [0.11]	0.78 ± 0.05	0.80 [0.07]	0.05 (−0.17; 0.16)	0.719	0.25 (−0.58; 0.83)	Small
Left kidney	0.74 ± 0.12	0.68 [0.11]	0.76 ± 0.05	0.75 [0.06]	0.05 (−0.17; 0.17)	0.629	0.33 (−0.52; 0.85)	Medium
Intestines	16.86 ± 1.02	17.37 [0.92]	17.42 ± 2.53	16.25 [1.36]	−0.41 (−1.56; 5.51)	1.000	0.00 (−0.73; 0.73)	Trivial

IQR: interquartile range. SD: standard deviation. CI: confidence interval. g: grams. ^*∗*^*p* values from the Mann–Whitney *U* test. *r*_*B*_: rank-biserial correlation.

## Data Availability

The data used to support the findings of this study are available from the corresponding author upon reasonable request.

## Abbreviations

AEBF: Aqueous extract obtained from the root bark of the medicinal plant *Bredemeyera floribunda* Willd
ARRIVE 2.0: Animal research: reporting of in vivo experiments
AKT: Serine/threonine kinase
BM: Body mass
CEUA/UFMS: Ethics Committee on the Use of Animal of Federal University of Mato Grosso do Sul/Comitê de ética no Uso de Animais
CEMTEC: Mato Grosso do Sul Weather and Climate Monitoring Center Centro de Monitoramento do Tempo e do Clima de Mato Grosso do Sul
CG: Control group
CGMS: Herbarium of Federal University of Mato Grosso do Sul
CONCEA: National Council for the Control of Animal Experimentation/Conselho Nacional de Controle de Experimentação Animal
EG: Extract group
JASP: Jeffreys's amazing statistics program
LD_50_: Medium lethal dose
OECD: The Organization for Economic Cooperation and Development
WHO: World Health Organization
PI3k: Phosphoinosotide 3-kinases
SisGen: National Genetic Heritage Management System/Sistema nacional de gestão do patrimônio genético.
